# Integrative bioinformatics approaches to establish potential prognostic immune-related genes signature and drugs in the non-small cell lung cancer microenvironment

**DOI:** 10.3389/fphar.2023.1153565

**Published:** 2023-04-03

**Authors:** Jiao Zhou, Shan Shi, Yeqing Qiu, Zhongwen Jin, Wenyan Yu, Rongzhi Xie, Hongyu Zhang

**Affiliations:** ^1^ The Fifth Affiliated Hospital of Sun Yat-sen University, Zhuhai, China; ^2^ Zhongshan School of Medicine, Sun Yat-sen University, Guangzhou, China; ^3^ Cancer Center, The Fifth Affiliated Hospital of Sun Yat-sen University, Zhuhai, China

**Keywords:** non-small cell lung cancer, tumor microenvironment, estimate, prognostic gene signature, drug sensitivity

## Abstract

**Introduction:** Research has revealed that the tumor microenvironment (TME) is associated with the progression of malignancy. The combination of meaningful prognostic biomarkers related to the TME is expected to be a reliable direction for improving the diagnosis and treatment of non-small cell lung cancer (NSCLC).

**Method and Result:** Therefore, to better understand the connection between the TME and survival outcomes of NSCLC, we used the “DESeq2” R package to mine the differentially expressed genes (DEGs) of two groups of NSCLC samples according to the optimal cutoff value of the immune score through the ESTIMATE algorithm. A total of 978 up-DEGs and 828 down-DEGs were eventually identified. A fifteen-gene prognostic signature was established *via* LASSO and Cox regression analysis and further divided the patients into two risk sets. The survival outcome of high-risk patients was significantly worse than that of low-risk patients in both the TCGA and two external validation sets (*p-value* < 0.05). The gene signature showed high predictive accuracy in TCGA (1-year area under the time-dependent ROC curve (AUC) = 0.722, 2-year AUC = 0.708, 3-year AUC = 0.686). The nomogram comprised of the risk score and related clinicopathological information was constructed, and calibration plots and ROC curves were applied, KEGG and GSEA analyses showed that the epithelial-mesenchymal transition (EMT) pathway, E2F target pathway and immune-associated pathway were mainly involved in the high-risk group. Further somatic mutation and immune analyses were conducted to compare the differences between the two groups. Drug sensitivity provides a potential treatment basis for clinical treatment. Finally, EREG and ADH1C were selected as the key prognostic genes of the two overlapping results from PPI and multiple Cox analyses. They were verified by comparing the mRNA expression in cell lines and protein expression in the HPA database, and clinical validation further confirmed the effectiveness of key genes.

**Conclusion:** In conclusion, we obtained an immune-related fifteen-gene prognostic signature and potential mechanism and sensitive drugs underling the prognosis model, which may provide accurate prognosis prediction and available strategies for NSCLC.

## Introduction

Lung cancer remains one of the most threatening malignancies to human health worldwide with relatively high incidence and mortality (Sung et al., 2021). NSCLC is the predominant histological subtype, comprising approximately 85% of lung cancer (Travis et al., 2015). Surgical resection is recommended for early-stage NSCLC, and adjuvant platinum-based chemotherapy confers a 5-year survival benefit rate, which increased by 5% for stage II-IIIA disease (Arriagada et al., 2010). Because of the concealed pathogenesis, approximately 60% of patients with NSCLC have locally advanced or metastatic disease, and conventional chemoradiotherapy has become the optimal treatment (Osmani et al., 2018). However, with a high recurrence rate after pulmonary resection, there was a significant sensitivity difference and clear toxic effects of chemoradiotherapy. Owing to the molecular heterogeneity of NSCLC, patients show various responses to conventional therapies, and even standard therapy according to the guidelines has major limitations. Therefore, precise individualized medicine gradually replaced the traditional one-size-fits-all toxic treatment (Herbst et al., 2018).

Over the past decade, molecularly targeted therapies targeting driver gene abnormalities have dramatically changed the treatment strategy for NSCLC; however, these new targeted drugs also present insufficient therapy due to the development of tumor resistance (Denisenko et al., 2018). Anti-PD-1/PD-L1 immunotherapy demonstrates an overall survival benefit in advanced NSCLC, but only 20% of patients benefit from immune checkpoint inhibitors (Huang et al., 2018). In conclusion, although substantial and promising achievements have been made in the therapeutic strategy of NSCLC, the prevention, early detection and treatment of NSCLC are still challenging. Therefore, it is particularly crucial to continue to identify new target genes and therapies to improve the curative effect and outcomes of NSCLC patients.

Malignant solid tumor tissue consists of not only cancer cells but also the TME, which contains extracellular matrix, stromal cells, and immune cells (Binnewies et al., 2018). Studies have certified that the TME influences the gene expression of tumor tissues in a variety of ways and then facilitates the occurrence, progression and metastasis of tumors (Hu and Polyak, 2008). Immune cells and adaptive immune cells in the TME act directly on cancer cells or through cytokine and chemokine signaling to influence tumor behavior and therapeutic reactions (Schulz et al., 2019). By taking advantage of the negative regulatory mechanism in the human immune system, malignant tumor cells can generate wholescale immune suppression in the TME to counteract the body’s antitumor immune effect (Teng et al., 2015). The difference in individual efficacy in tumor immunotherapy is strongly associated with immunosuppression in the TME (Anari et al., 2018). Therefore, exploring new biomarkers related to the tumor microenvironment opens up new avenues for precise individualized therapy of NSCLC.

To understand the impact of the tumor genetic genome on clinical prognosis, comprehensive whole genome gene expression collections have been established (Blum et al., 2018). In addition, to predict the infiltration of non-tumor cells in tumor tissues, ESTIMATE algorithms have been designed to premeasure tumor purity utilizing gene expression information from The Cancer Genome Atlas (TCGA) database (Yoshihara et al., 2013). This algorithm was soon applied to ovarian cancer (Hornburg et al., 2021), renal cell carcinoma (Xu et al., 2019), and diffuse large B-cell lymphoma (Lou et al., 2022). Therefore, bioinformatics analysis based on TME-related prognostic signatures has become possible. In this study, the ESTIMATE algorithm and CIBERSORT algorithm in R language were utilized to explore the tumor microenvironment of patients with NSCLC in the TCGA database. First, we identified differentially expressed genes with prognostic and therapeutic value in the tumor microenvironment and predicted their regulatory network. Furthermore, we developed an innovative prognostic signature for risk stratification based on prognostic genes, and the potential biological mechanism and therapeutic drugs based on the prediction model were evaluated. This result provides additional prospective therapeutic interventions and personalized treatment strategies for NSCLC patients.

## Materials and methods

### Chip data acquisition and processing

We downloaded the gene expression data of NSCLC patients and related clinical materials meeting the study criteria from the TCGA databases (https://portal.gdc.cancer.gov/). Patients were enrolled when they met the following criteria: a) pathologically confirmed NSCLC; b) available detailed prognostic information; and c) complete mRNA expression data. A total of 955 NSCLC patients with stage I-IV disease were included through screening. The clinical information of patients included age, sex, survival status, overall survival, last follow-up time, T stage, N stage, M stage and clinical stage. The TCGA-NSCLC cohort was used as a training set to construct an immune score prognostic model. For further verification of the performance of immune scores in predicting survival, transcriptome sequencing profiles and corresponding clinical data of two other cohorts of NSCLC patients were obtained from the Gene Expression Omnibus (GEO) datasets (https://www.ncbi.nlm.nih.gov/), namely, GSE31210 and GSE37745. GSE31210 comprised 226 early-stage NSCLC patients, and GSE37745 included 196 NSCLC samples. GSE31210 and GSE37745 were downloaded based on Affymetrix U133 Plus 2.0.

### ESTIMATE algorithm and identification of stromal and immune groups

Based on R statistical software (version 4.1.0; https://www.r-project.org/), the proportion of stromal and immune cells in each tumor tissue sample was calculated by the ESTIMATE algorithm, and the ratio was represented in the form of immune, stromal and ESTIMATE scores. The stromal score represented the percentage of stromal cells in the TME, the immune score was used to assess the ratio of immune cells, and the ESTIMATE score represented the comprehensive level of the immune and matrix score. The Surv_cutpoint function was used to find the best cutoff values for the immune, stromal and ESTIMATE scores. NSCLC patients were categorized into high- and low-score groups based on the optimal cutoff value of the related score. The “survival” and “survminer” packages were utilized to evaluate the overall survival of NSCLC patients on the basis of the high-low score, which included the immune, stromal and ESTIMATE scores. Associations of the abovementioned scores with clinicopathologic characteristics were also further assessed by unpaired t-tests.

### DEGs screening and functional enrichment analysis

The DEG screening between the high and low immune score groups was constructed through the “DESeq2” R package (version 4.1.0), and a false discovery rate (FDR) < 0.05 and |log2-fold change| (|log2FC|) ≥ 1 were set up to screen DEGs. A higher gene expression value was selected if multiple probes measured the same gene. The selected DEGs were visualized through the “ggplot2” package of R to generate scatter plots and heatmaps.

Functional enrichment analyses, including molecular function (MF), cell component (CC), and biological process (BP), were performed for the DEGs using the “cluster-Profiler” package. The “cluster-Profiler” package was used to analyze the Kyoto Encyclopedia of Genes and Genomes (KEGG), which was used to identify the crucial signal pathways between upregulated and downregulated DEGs. Terms were identified as statistically significantly enriched with the threshold of *p-value* < 0.05 for gene ontology (GO) and KEGG. In addition, GO and KEGG analyses were evaluated by fold enrichment scores to determine which genetic functions and cell signaling pathways may be relevant to DEGs.

### Prognostic DEGs screening

First, univariate Cox analysis using the “survival” package was employed to screen prognostic DEGs that were significantly relevant to the overall survival (OS) of 936 NSCLC patients in the TCGA cohort. The candidate prognostic DEGs with *p-value* < 0.01 were used for the subsequent analysis. Subsequently, the candidate prognostic DEGs underwent least absolute shrinkage selection operator (LASSO) analysis. Eventually, multivariate Cox regression analysis was conducted to calculate the hazard ratios (HRs) with a 95% confidence interval (95% CI) and determine prognostic genes.

### Establishment and evaluation of prognostic signature

Each NSCLC patient’s survival risk score in the TCGA cohort was calculated according to the mRNA expression of optimal prognostic genes multiplied by the corresponding regression coefficients.

$$The\;computational\;formula\;is\;as\;follows:risk\;score=\sum_{n=1}^{j}Coef\;j*Xj$$

Coef j is the regression coefficient determined through multivariate Cox analysis, and Xj refers to the normalized mRNA expression level of optimal genes. NSCLC patients in the TCGA were separated into high- and low-risk subtypes according to the median threshold of the risk score.

The OS between the two risk groups was evaluated by Kaplan-Meier analysis with the log-rank test. In addition, external validation of the GSE31210 and GSE37745 datasets was performed to predict the accuracy of the prognostic DEG signature. The Time-dependent ROC curves were performed for confirmingthe prognostic DEG signatur e’s prognosis capability by calculating the AUC of the 1-, 2-, and 3-year OS of NSCLC patients in TCGA. Finally, the GSE31210 and GSE37745 datasets were used for external validation to corroborate the results.

### Construction of predictive nomogram

First, univariate and multivariate Cox regression analyses were utilized to evaluate the individual covariates, the risk score calculated above and the associated clinicopathological parameters, which clearly impacted the patient’s survival outcome. A *p-value* < 0.05 was considered to be the significance threshold. Then, the nomogram was constructed through calibration plots to predict 1-, 3- and 5-year overall survival, using the concordance index (C-index) to test internal validation. The predictive value of the nomogram, risk score and other clinical parameters were compared by ROC curves.

### Pathway enrichment analysis

KEGG was performed to explore signal pathways between the high- and low-risk groups. DEGs underwent gene set enrichment analysis (GSEA), which aimed to better confirm the molecular and biological mechanisms between the two groups.

The pathway enrichment of differences underlying gene sets in two risk score subgroups was calculated by the annotation file “hallmark gene sets” of the Molecular Signatures Database (MSigDB) (https:// www.gseamsigdb.org/gsea/msigdb/).

### Immune infiltration assessment

The CIBERSORT algorithm was applied to evaluate the relative infiltration abundance of various types of tumor-infiltrating immune cells. We utilized CIBERSORT through an online R script in the local R environment, and the algorithm was iterated with 1,000 permutations and based on the LM22 gene signature. Related results were filtered with a *p-value* < 0.05. Twenty-two subtypes of immune cells between the two risk groups were subsequently compared. As supplementary, the mRNA expression of typical immune checkpoints between the two groups was further compared.

### Mutation analysis and prediction of the sensitive drugs

Mutation information of NSCLC was retrieved from the TCGA database. The Mutation Annotation Format (MAF) form was used to reserve somatic variant data. Maftools of the R package was used to identify the top 30 most frequently mutated genes between the two risk cohorts. The “pRRophetic” R package was utilized to estimate the IC50 of common drugs between the patients in the two groups using the Wilcoxon signed-rank test to predict the drug sensitivity of the groups.

### Exploration of hub genes in prognosis signature and experimental validation

We first built a protein-protein interaction (PPI) network to examine the interplay between the prognostic DEGs obtained from univariate Cox regression analysis. Then, the DEGs were uploaded to String (https://string-db.org/), and the PPI network was constructed and further visualized and analyzed by Cytoscape software (version 3.8.0).

Molecular Complex Detection (MCODE) screened the modules of the subnetwork, and the cutoff criterion was degree cutoff = 2, node score cutoff = 0.2, k-core = 2 and max depth = 100. The common gene, overlapping result from PPI and multiple cox analysis, was selected as the key prognosis gene. First, the protein expression levels of tumor or normal tissue were confirmed based on the HPA (The Human Protein Atlas) database (https://www.proteinatlas.org). The “Survival” package was used to assess the prognosis among the normalized mRNA expression of the prognostic hub genes. Kaplan-Meier analysis was used for evaluating the impact of quantities of gene expression on patient survival according to individual mRNA expression of particular prognostic genes (high versus low expression). Finally, PCR experiments were conducted to determine the mRNA expression of hub genes in normal lung epithelial cells and NSCLC cell lines.

### Real-time RT-PCR

The total RNA of relative cell lines was extracted using the RNeasy mini kit following the manufacturer’s protocol (Qiagen). RNA was eluted in 30 μL of RNase-free water and stored at −80 ℃ . RNA (500 ng) was reverse-transcribed using the PrimeScript™ RT reagent kit (Takara Bio, Inc., Otsu, Japan). Then, PCR amplification of the cDNA was performed using TB Green^®^ Premix Ex Taq™ II (Takara Bio, Inc.) according to the manufacturer’s instructions. The sample volume was 10 μL , and the following reaction conditions were used: 95°C for 30 s (predenaturation), then 40 cycles at 95°C for 10 s (denaturation), 55°C for 30 s (annealing), 72°C for 30 s (extension) and the ultimate extension at 72°C. Relative mRNA expression levels were acquired by the $2^{-∆∆Cq}$ method.

Gene primer sequences were acquired from Generay Biotechnology (Shanghai, China). Table 1 describes the detailed sequences.

**TABLE 1:** Primers sequences corresponding to prognostic genes and GAPDH.

Genes name		(5′to3′)
ADH1C	Forward primer	CTC​GCC​CCT​GGA​GAA​AGT​C
	Reverse primer	GGC​CCC​CAA​CTC​TTT​AGC​C
EREG	Forward primer	GTG​ATT​CCA​TCA​TGT​ATC​CCA​GG
	Reverse primer	GTG​ATT​CCA​TCA​TGT​ATC​CCA​GG
GAPDH	Forward primer	GGA​GCG​AGA​TCC​CTC​CAA​AAT
	Reverse primer	GGC​TGT​TGT​CAT​ACT​TCT​CAT​GG

### Statistical analysis

The distributional differences in clinical variables between the two risk sets were analyzed by the chi-square test. Univariate and multivariate Cox regression analyses were adopted to assess independent prognostic parameters, and HRs and 95% CIs were evaluated at the same time. The Kaplan-Meier method was applied to generate survival curves for prognosis analyses, and the log-rank test was used to define the significance of differences. Statistical analyses in the study were conducted by R software (version 4.1.0), IBM SPSS Statistics (version 25.0) or GraphPad Prism (version 8.0). If not mentioned above, a threshold of *p-value* < 0.05 was defined as statistical significance.

## Results

### Flow of data collection and analysis

**FIGURE 1:**
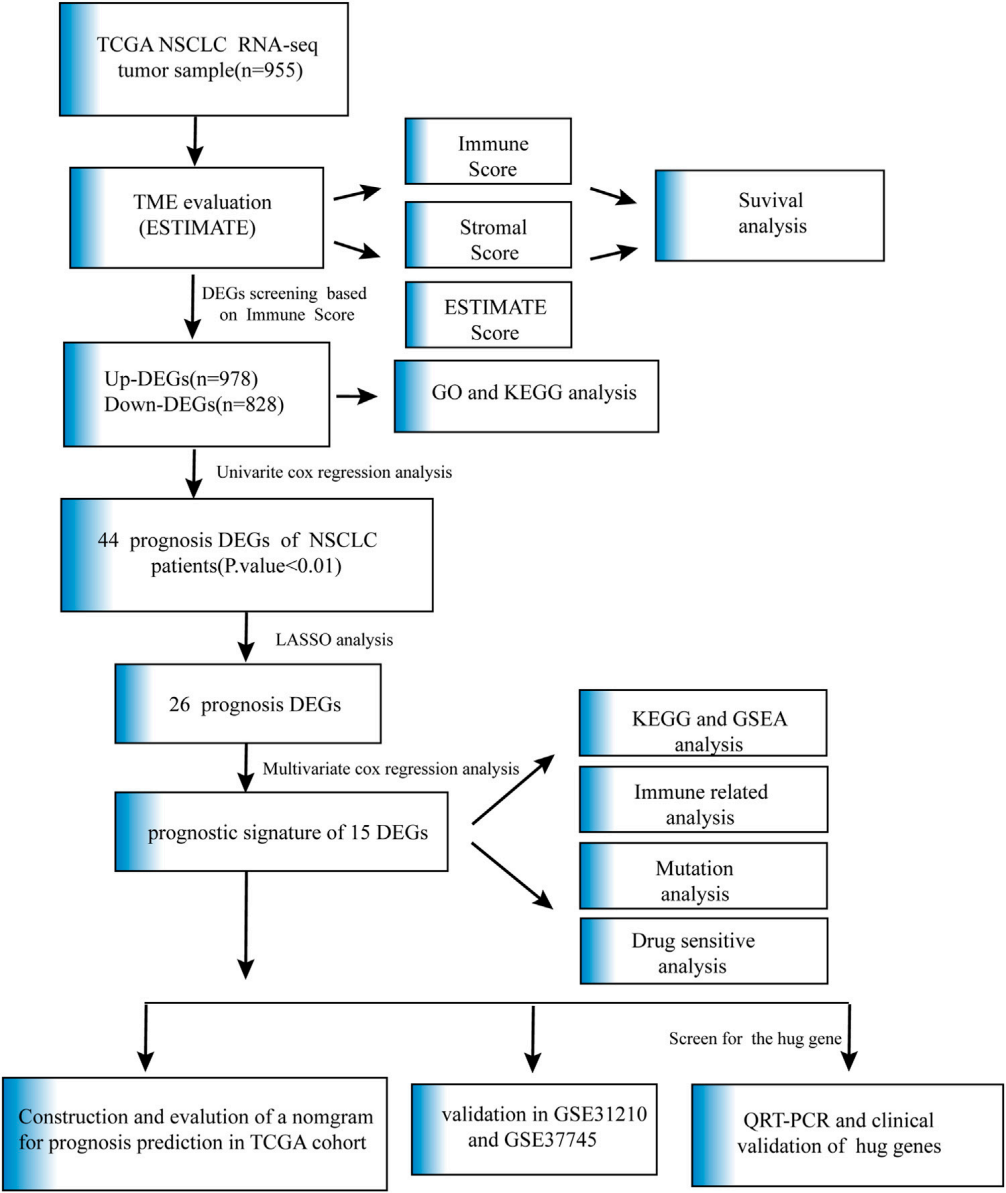
Flow chart of data collection and analysis.

In our research, we utilized ESTIMATE algorithms to calculate the immune, stromal and ESTIMATE scores in NSCLC patients after obtaining mRNA expression profiles and corresponding clinical characteristics from the TCGA cohort. By comparing the relationship between each score and survival outcome together with clinicopathological parameters, the immune score was shown to play a vital role in the prognosis of NSCLC patients. We identified the immune-related DEGs based on high- and low-immune score subtypes and predicted their potential biological functions and pathways. The DEGs associated with OS were analyzed and screened by univariate Cox analysis, and the genes with *p-value* ≤ 0.01 were further analyzed by LASSO analysis and subsequent multivariate Cox analysis. A 15-gene prognostic signature was developed. A gene-based classifier was generated, and NSCLC samples in our study were classified into two risk cohorts based on the median risk score obtained from the risk score computational formula. OS was evaluated by Kaplan-Meier analysis between the two groups. The nomogram integrated risk score and related clinical information, and calibration plots and ROC curves were applied to verify prognosis accuracy. The GSE31210 and GSE37745 datasets, as external validations, also confirmed the high predictive efficiency of the 15-gene diagnostic model described above. KEGG and GSEA were performed to explore the molecular and biological differences, and further mutation and drug sensitivity analyses were also conducted between the patients in the two risk groups. In addition, immune-related analysis was performed to compare the proportions of immune cells in the high- and low-risk groups. Finally, the common gene, selected as the key prognostic gene of two overlapping results from PPI and multiple Cox analyses, was verified by comparing the mRNA expression of normal lung epithelial and NSCLC cell lines and protein expression in the HPA database. The clinical validation of the TCGA database further confirmed the effectiveness of key genes. Details are described in Figure 1.

### Immune score was connected with the prognosis of NSCLC patients

**FIGURE 2:**
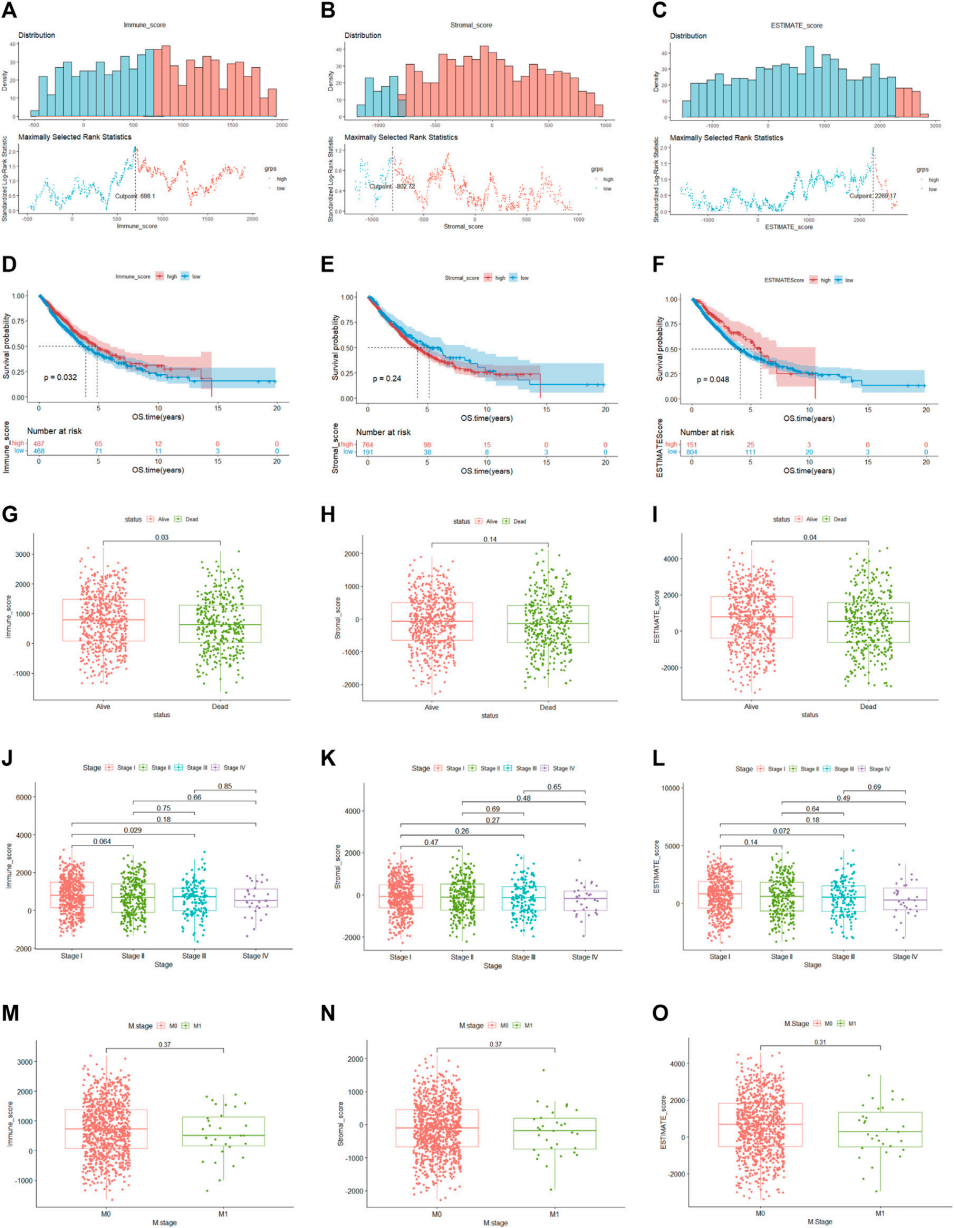
Relationship between clinical characteristics and immune, stromal and ESTIMATE scores **(A–C)**. The optimal cutoff values of the immune, stromal and ESTIMATE scores. **(D–F)**. **(K–M)** analysis of immune, stromal and ESTIMATE scores. **(G–I)**. Distribution of the three scores among patients with different statuses **(J–L)**. Distribution of immune, stromal and ESTIMATE scores among NSCLC stages **(M–O)**. Distribution of three scores between M-stage of NSCLC.

Among 955 NSCLC cases enrolled in our study in the TCGA cohort, 489 were LUAD and 466 were LUSC patients. According to the ESTIMATE algorithm, the generated immune, stromal and ESTIMATE scores were utilized in the Kaplan–Meier survival analysis. The best demarcation values of the abovementioned thresholds for subsequent survival analysis were 698.1, −802.72 and 2,296.17, respectively (Figures 2A–C). The results in our research demonstrated that a higher immune and ESTIMATE score was associated with a better prognosis (*p* < 0.05) (Figures 2D, F); however, there was no significant correlation with the stromal score (*p* = 0.24) (Figure 2E). The three scores were subsequently analyzed to evaluate the relationship with clinicopathologic characteristics (Figures 2G–O), showing that living NSCLC patients had notably higher immune and ESTIMATE scores (*p* < 0.05), and the immune score declined along with the progression of stage and M stage classification despite no striking difference. In contrast, the stromal score was not associated with the abovementioned clinical features. These findings clarified that the immune and ESTIMATE scores played a crucial role in the progression of NSCLC. Since the ESTIMATE score represents a combination of immune and stromal scores, the immune score seemed to be a better indicator of prognosis in NSCLC patients.

### DEG screening and functional analysis between low- and high-immune score groups

**FIGURE 3:**
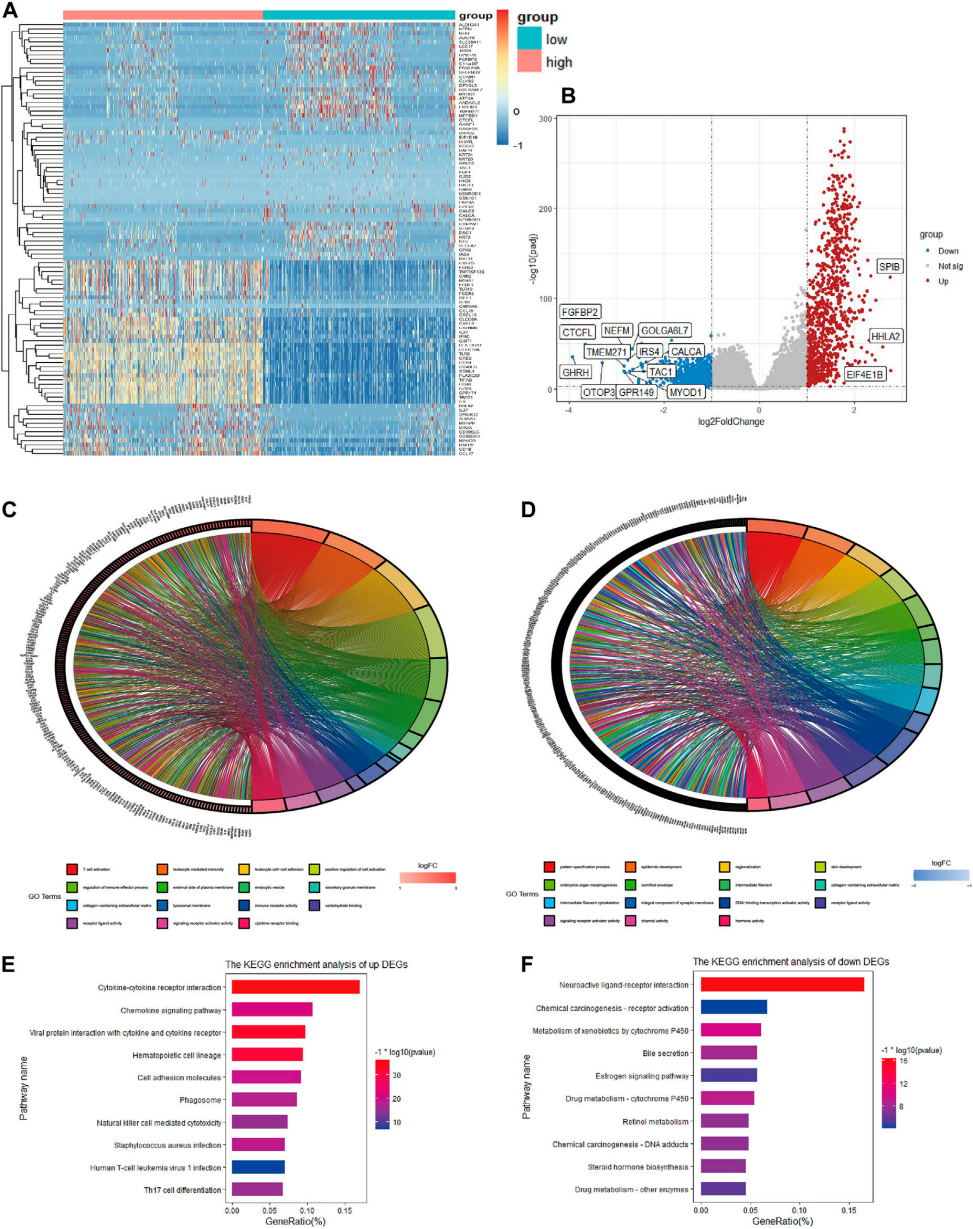
Heatmap, volcano plot and enrichment analysis of GO and KEGG for DEGs **(A)**. Heatmap of DEGs in TCGA **(B)**. Volcano plot of DEGs in TCGA **(C)**. Top 5 enriched biological processes, molecular functions, and cellular components of upregulated co-DEGs and **(D)** downregulated co-DEGs **(E)**. Top 10 KEGG pathways of upregulated co-DEGs and **(F)** downregulated co-DEGs.

To determine the global gene expression profiles in the high and low immune score groups, DEG analysis was further conducted. As a result, 1806 DEGs containing 978 upregulated and 828 downregulated genes were determined. The expression distribution of DEGs was visualized by volcano plots (Figure 3B), and the top 50 DEGs of the two groups are illustrated in the heatmap (Figure 3A). Moreover, potential biological function analysis was conducted, and the top 5 GO annotations (BP, CC, MF) of up- and downregulated DEGs are described in the circle plot of Figures 3C, D. As shown, the upregulated DEGs were primarily linked to immune functions, including T cell activation, immune receptor activity and lymphocyte activation regulation, while pattern specification process, epidermis development and DNA-binding transcription activator activity were enriched in downregulated DEGs. Furthermore, KEGG analysis demonstrated that the upregulated DEGs were significantly associated with cytokine-cytokine receptor interactions, cell adhesion molecules and chemokine signaling pathways (Figure 3E). However, the downregulated DEGs were enriched in neuroactive ligand-receptor interactions and chemical carcinogenesis-receptor activation (Figure 3F).

### Screening of independent prognostic feature DEGs

**FIGURE 4:**
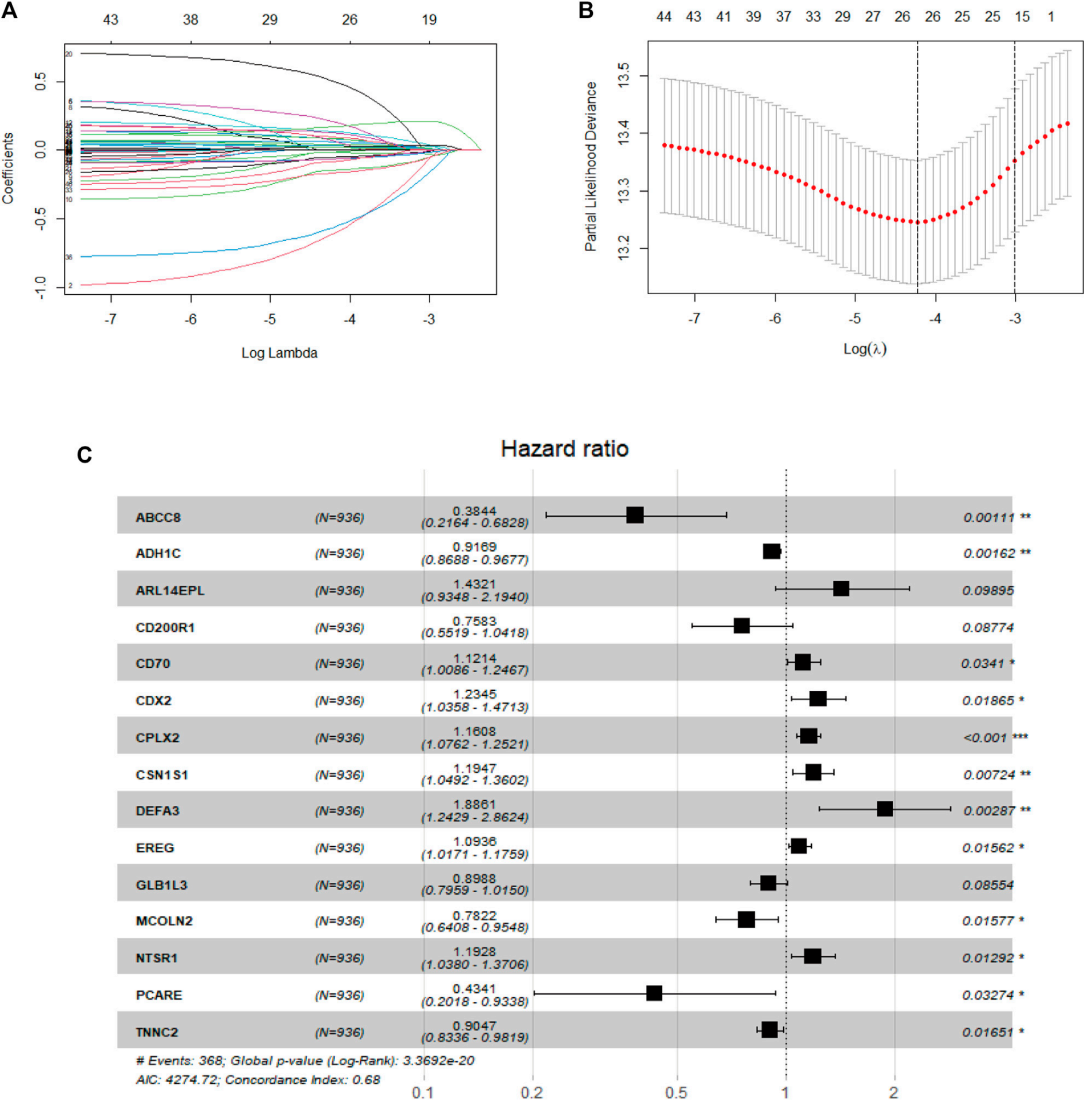
Development of the prognostic signature in the TCGA cohort **(A)**. Diagnostic model construction using a LASSO regression model **(B)**. Coefficient distribution plots to select the optimum lambda value **(C)**. Results of multivariate Cox regression analysis of OS in the TCGA cohort.

In the beginning, univariate Cox analysis was utilized to analyze the 1806 DEGs selected from this study, and genes with a *p-value* < 0.01 were incorporated in survival-related analysis and used for subsequent LASSO and further multivariate Cox analysis. Twenty-six DEGs were screened through LASSO analysis and were significantly correlated with survival outcome (Figures 4A, B). After subsequent screening, the results of multivariate Cox analysis revealed that a prognostic signature of 15 genes was independently related to OS, including alcohol dehydrogenase 1C (ADH1C), complexin2 (CPLX2), casein alpha s1 (CSN1S1), neurotensin receptor 1 (NTSR1), caudal type homeobox 2 (CDX2), ATP binding cassette subfamily C member 8 (ABCC8), photoreceptor cilium actin regulator (PCARE), troponin C2, fast skeletal type (TNNC2), epiregulin (EREG), mucolipin TRP cation channel 2 (MCOLN2), CD70 molecule (CD70), galactosidase beta 1 like 3 (GLB1L3), CD200 receptor 1 (CD200R1), defensin alpha 3 (DEFA3), and ADP ribosylation factor like GTPase 14 effector protein like (ARL14EPL). These genes were represented by a forest map (Figure 4C). Supplementary information is described in Supplementary Table S1.

### Establishment and validation of prognostic model

The prognostic signature was identified, and the risk score of each NSCLC patient was calculated using the following formula:

The risk score = (−0.08672) × *Exp*ADH1C + (0.14913) × *Exp* CPLX2 + (0.17786) × *Exp*CSN1S1 + (0.17628) × *Exp*NTSR1 + (0.21064) × *Exp*CDX2 + (−0.9561) × *Exp* ABCC8 + (−0.83455) × *Exp*PCARE + (−0.10013) × *Exp*TNNC2 + (0.08950) × *Exp* EREG + (−0.24566) × *Exp* MCOLN2 + (0.11454) × *Exp* CD70 + (−0.10665) × *Exp* GLB1L3 + (−0.27673) × *Exp* CD200R1 + (0.63454) × *Exp* DEFA3 + (0.35913) × *Exp* ARL14EPL.

**FIGURE 5:**
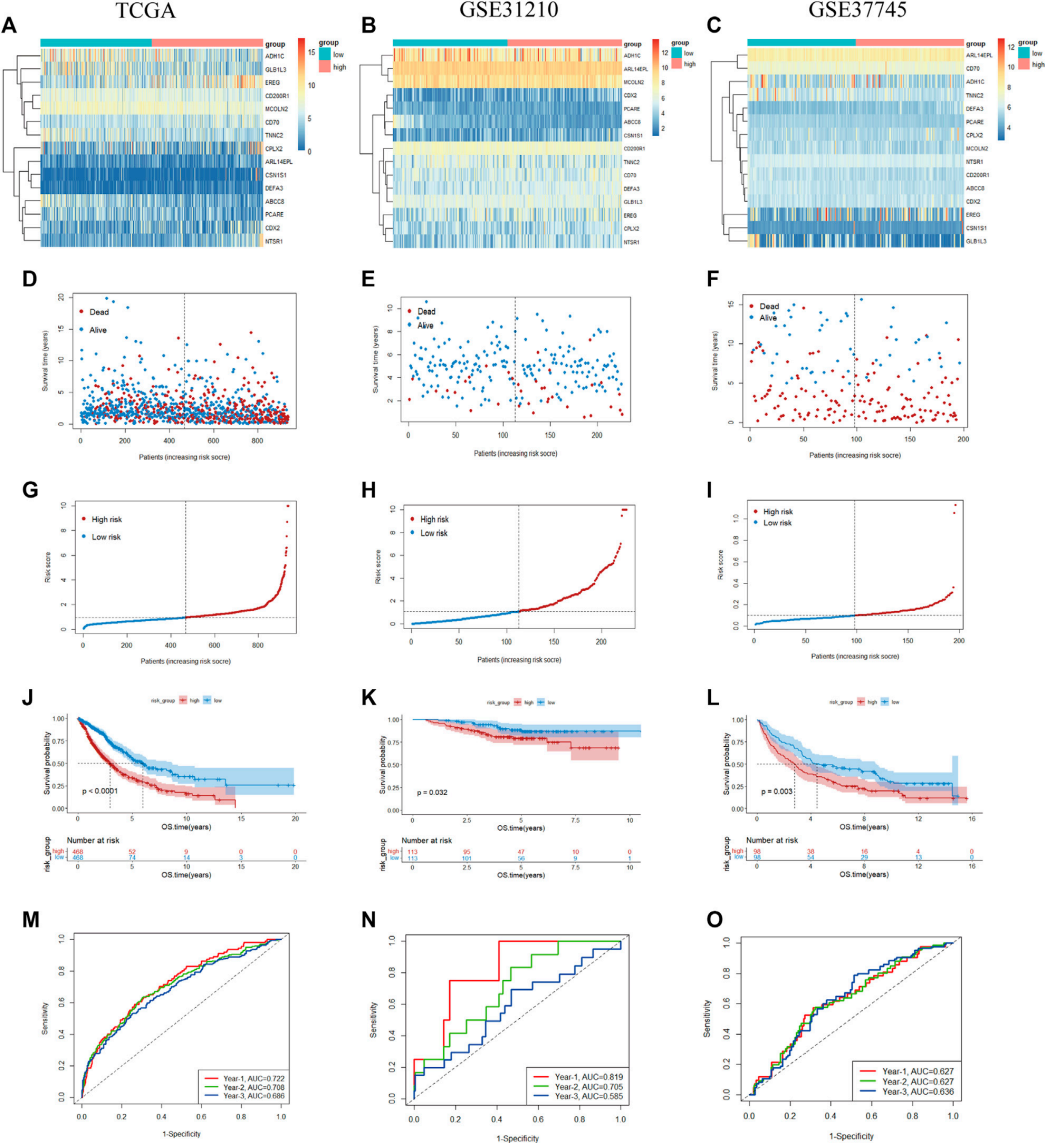
Prognostic value of the 15-gene prognostic model in the TCGA and validation cohorts **(A–C)**. Heatmap of fifteen genes between the two groups in the TCGA and validation sets **(D–F)**. Risk score scatter plot. Red dots indicate dead patients, and blue dots indicate alive patients **(G–I)**. Risk score curve plot. The dotted line indicates the individual distribution of the risk score, and the patients are categorized into low-risk (blue) and high-risk (red) groups **(J–L)**. Survival status and time of patients between the two groups in the TCGA and validation sets, respectively **(M–O)**. The time-dependent ROC curve of patients between the two groups in the TCGA and validation sets.

Based on the demarcation point of the risk score, the NSCLC samples were divided into high- or low-risk categories. The mRNA expression of 15 genes in the two risk subtypes was presented in the form of a heatmap (Figure 5A). The patients had a higher risk of death with increasing risk score, as shown in the risk score curve and scatter plot (Figures 5D, G). Subsequently, the K-M analysis revealed that the prognosis of NSCLC samples in the low-risk subgroup was remarkably better than that in the high-risk subgroup (*p* < 0.05) **(** Figure 5J). For completeness, the time-dependent ROC curves predicted the OS of NSCLC patients (AUC of 1-year = 0.722, 2-year = 0.708, 3-year = 0.686) **(** Figure 5M **)**.

To validate the reliability of our prognostic signature constructed from the TCGA cohort, the risk score was further calculated with the abovementioned formula for each patient in GSE31210 (226 LUADs) and GSE37745 (n = 196, 106 LUADs, 24 LCLCs, 66 LUSCs). Patients were also split into high- and low-risk subtypes according to the cutoff point of the risk score. The heatmap shows the expression of 15 prognostic genes in the two risk groups (Figures 5B, C). Similar to the results of TCGA, patients tended to have a higher probability of death in the high-risk group than in the low-risk group (Figures 5E, F, H, I). As a supplementary, we evaluated the prognostic value of prognostic features in LUAD and LUSC, validation in the NSCLC subtypes also demonstrated the similar result (Supplementary Figure S1). We also observed a significant OS difference in GSE31210 and GSE37745, which also implied the prognostic value of the gene signature (*p* < 0.05) (Figures 5K, L). As shown in Figures 5N, O **,** ROC curves also reached preferable AUC values in the two validation sets, demonstrating the potent capability of the 15-gene prognostic model.

### Relationship between the risk score and clinical parameters

A total of 936 NSCLC patients (475 LUADs and 461 LUSCs) with complete clinical information were enrolled in our clinical prognostic analysis. Table 2 shows the association between the two risk groups and clinical factors, including age, sex, stage, T stage, smoking index, status and tumor type. Our study revealed that the high-risk group had a higher rate of males and LUSC patients and more advanced cases, while other clinical variables were not significantly associated with risk scores.

**TABLE 2:** Relationship between risk score and clinical characteristics of 936 patients in TCGA cohort.

Characteristics	Total *n* = 936 (%)	High-risk group *n* = 468 (%)	Low-risk group *n* = 468 (%)	*χ* ^*2*^	*p*-value
Age, year				0.852	0.356
≤ 65	408 (43.6%)	211 (45.1%)	197 (42.1%)		
>65	528 (56.4%)	257 (54.9%)	271 (57.9%)		
Gender				36.84	<0.001
Male	561 (59.9%)	326 (69.7%)	235 (50.2%)		
Female	375 (40.1%)	142 (30.3%)	233 (49.8%)		
Stage				6.816	0.009
Stage I/II	748 (79.9%)	358 (76.5%)	390 (83.3%)		
Stage III/IV	188 (20.1%)	110 (23.5%)	78 (16.7%)		
Smoking index				1.551	0.213
High	455 (48.6%)	369 (78.8%)	353 (75.4%)		
Low	481 (51.4%)	99 (21.2%)	115 (24.6%)		
Tumor type				33.86	<0.001
LUAD	475 (50.9%)	193 (41.2%)	282 (60.3%)		
LUSC	461 (49.3%)	275 (58.8%)	186 (39.7%)		

Abbreviations: n, number.

Similarly, as external validation, the results of GSE31210 indicated that the risk score was remarkably associated with EGFR mutation and had no association with other clinical parameters (Supplementary Table S2). In addition, GSE37745, also used for external validation, showed that the risk score was not associated with clinical parameters except tumor type (details are described in Supplementary Table S3).

### Screening of independent prognostic parameters and establishment of nomogram

**FIGURE 6:**
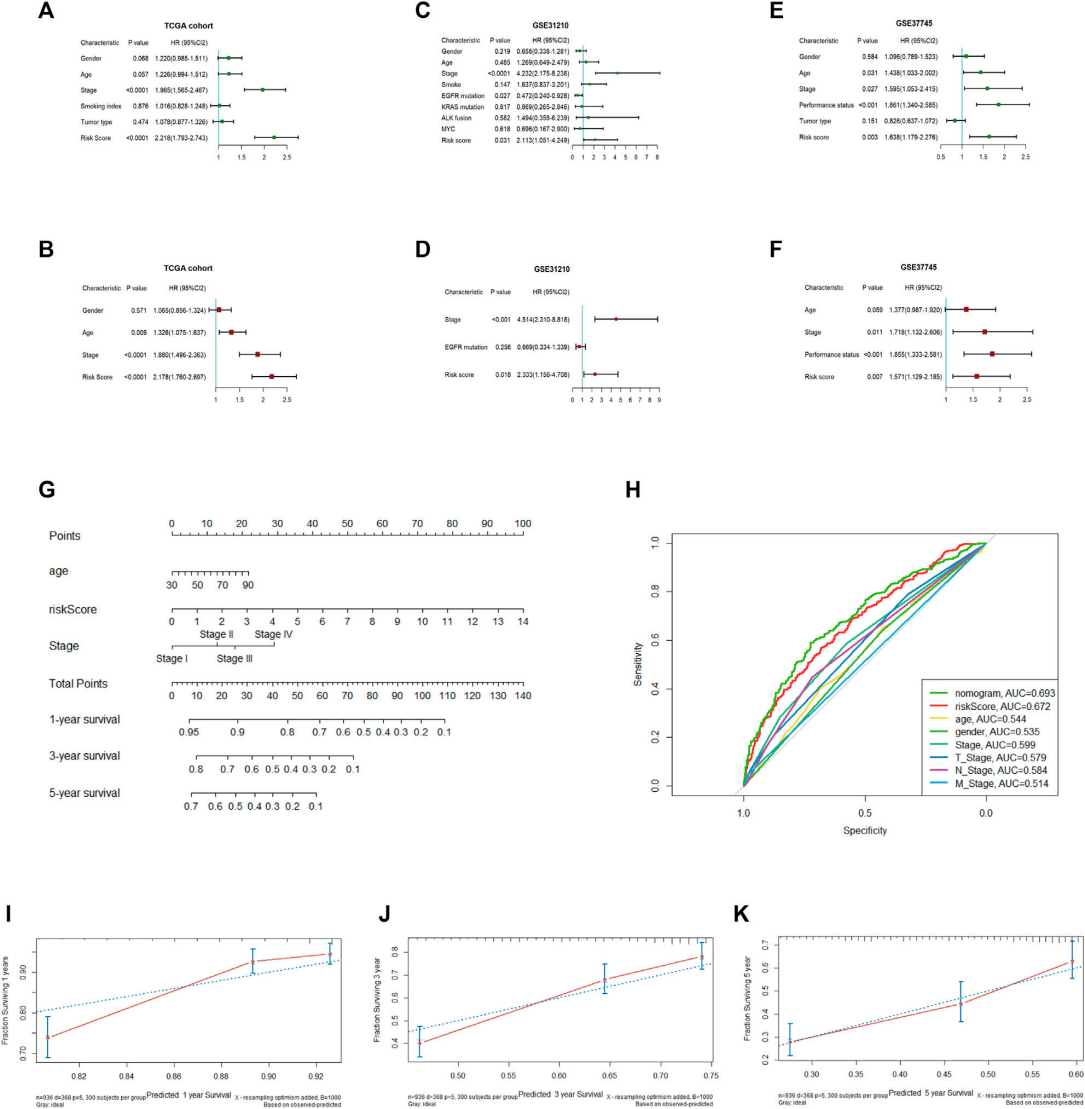
Univariate and multivariate Cox regression analyses in the TCGA and validation cohorts and establishment of the nomogram **(A, B)**. Univariate and multivariate Cox regression analyses in TCGA **(C, D)**. Univariate and multivariate Cox regression analyses in GSE31210 **(E, F)**. Univariate, multivariate Cox regression analysis in GSE37745 **(G)**. Establishment of a nomogram predicting OS based on the independent prognostic factors in TCGA **(H)**. ROC curve of the nomogram, risk score and other relevant clinical parameters in TCGA **(I–K)**. Calibration curves of the nomogram prediction of 1-, 3-, and 5-year survival in TCGA.

To assess whether the risk score and which clinical parameters could serve as independent predictive factors, subsequently, univariate and multivariate Cox analyses were conducted. A total of 936 NSCLC samples were enrolled from the TCGA cohort, as shown in Figures 6A, B. From the results of univariate Cox analysis, the stage (*p* < 0.0001) and risk score (*p* < 0.0001) were observably related to OS. We further chose the variable with a *p*-value < 0.1 for the multivariate Cox test, and the obtained results showed that the risk score (HR = 2.178; 95% CI, 1.760–2.697; *p* < 0.0001) remained significant for overall survival in NSCLC patients. In addition, stage (HR = 1.880; 95% CI, 1.496–2.363; *p* < 0.0001) and age (HR = 1.326; 95% CI, 1.075–1.637; *p* = 0.009) were also significant predictors of prognosis.

To demonstrate the accurate prediction efficiency of the prognostic signature that we established, univariate and multivariate Cox analyses were also performed in GSE31210 and GSE37745. The obtained results also indicated that the risk score (HR = 2.333; 95% CI, 1.156–4.708; *p* = 0.018) was correlated with survival in GSE31210 and could also be a good predictor in GSE37745 (HR = 1.571; 95% CI, 1.129–2.185; *p* = 0.007). The details are described in Figures 6C–F. Moreover, we constructed a nomogram based on three independent prognostic indices (age, stage and risk score) from TCGA (Figure 6G). The calibration curve, which evaluated the conformance of the nomogram, displayed high consistency between the nomogram-predicted probability and actual 1-, 3-, and 5-year OS (Figures 6I–K). The ROC curve showed prediction efficiency of the prognostic model (AUC = 0.693), and the risk score displayed better predictive efficiency than other clinical parameters (Figure 6H).

### Pathway enrichment analysis based on the prognosis model

**FIGURE 7:**
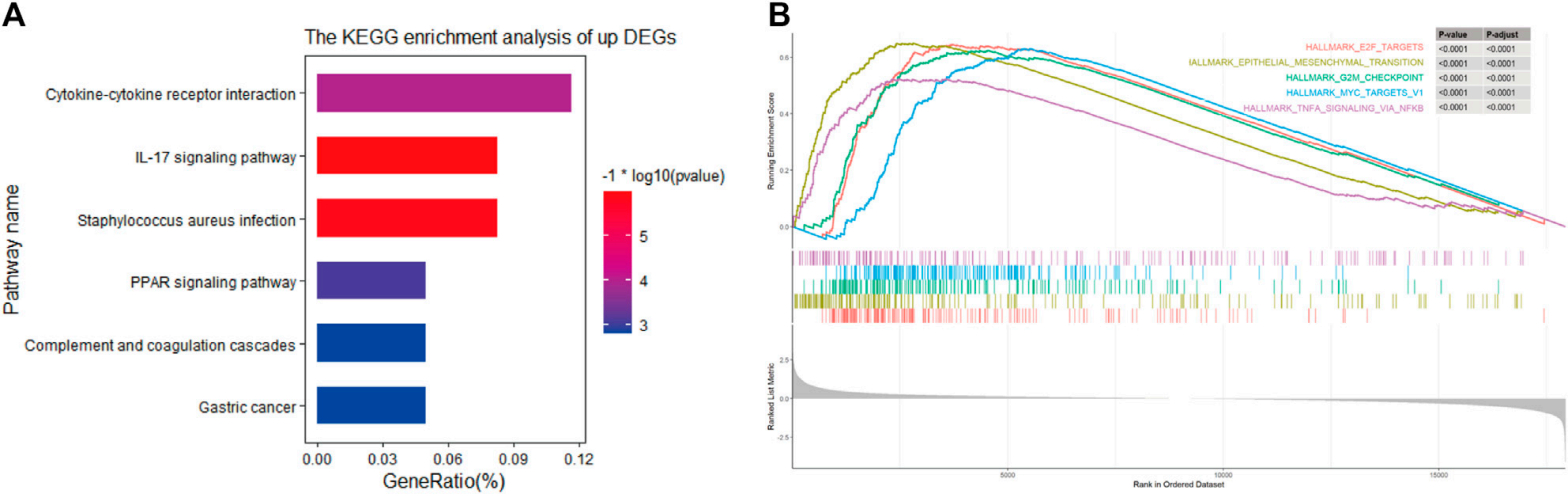
**(A)** KEGG pathways in high-risk group **(B)**. Top 5 Gene Set Enrichment Analysis of gene set of high-risk in TCGA cohort.

To further understand the relevant pathway mechanism between the two subgroups. KEGG pathway analysis and GSEA were performed on the DEGs of the high-risk groups, and the obtained results suggested that the signaling pathways of the high-risk subgroup were primarily enriched in the cytokine–cytokine receptor interaction pathway, IL-17 signaling pathway, PPAR signaling pathway and so on (Figure 7A). Additionally, GSEA indicated that the top 5 enriched pathways were epithelial mesenchymal transition (*P* adjust < 0.0001), E2F targets (*P* adjust < 0.0001), G2M checkpoint (*P* adjust < 0.0001), MYC targets (*P* adjust < 0.0001) and TNF-α signaling *via* NFKB (*P* adjust < 0.0001); details are presented in Figure 7B.

### Tumor immune related and mutation analysis of two subgroups

**FIGURE 8:**
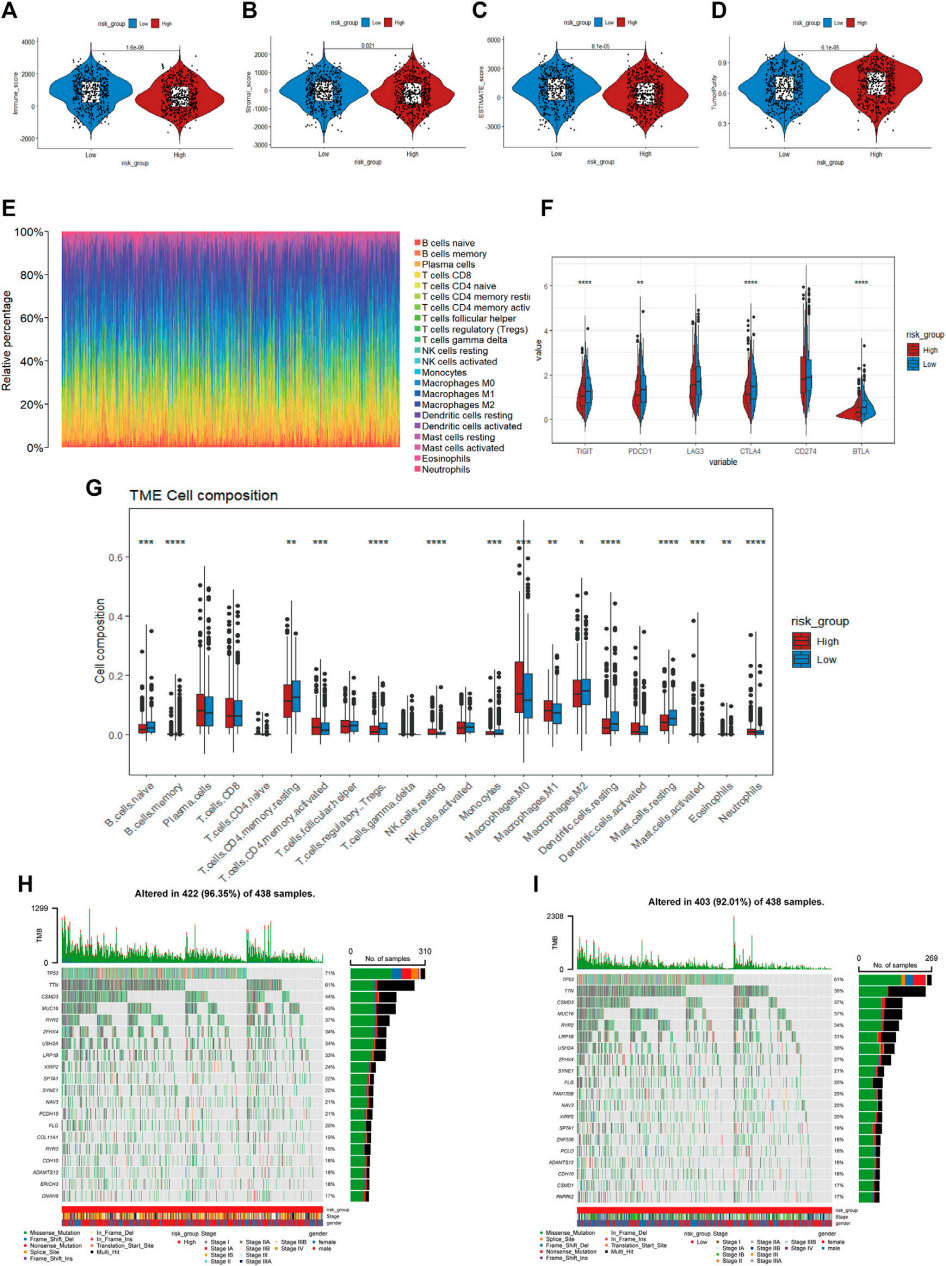
**(A–D)** Immune score, stroma score, ESTIMATE score and tumor purity of the high- and low-risk groups **(E)**. Distribution of infiltration of 22 immune cell types in the two risk groups **(F)** The expression of immune checkpoint genes between the two cohorts **(G)**. The proportions of different immune cells in the high- and low-risk groups **(H, I)**. Mutated gene mutation profiles between the high- and low-risk groups.

According to the results of KEGG and GSEA, we found that the risk score was linked to immunity; thus, the tumor immune microenvironment in the two risk categories was compared. The obtained results revealed a lower immune score (*p* < 0.001), stromal score (*p =* 0.021), and ESTIMATE score (*p* < 0.001) and a higher tumor purity (*p* < 0.001) in the high-risk group based on the ESTIMATE algorithm (Figures 8A–D). Additionally, the distribution of 22 infiltrating immune cells in the two risk groups was evaluated based on the CIBERSORT algorithm (Figure 8E), and it indicated a higher proportion of CD4 memory-activated T cells, NK cells, M0 and M1 macrophages and neutrophils in the high-risk group, whereas B cells, CD4 memory resting T cells, and Tregs accounted for more cells in the low-risk group (Figure 8G). We also compared the differential expression of immune checkpoint genes between the two cohorts (Figure 8F), such as PDCD1, TIGIT, CTLA4, and BTLA, which demonstrated remarkably lower mRNA expression in the high-risk group. Furthermore, we explored the top 20 mutated gene mutation profiles between the high- and low-risk groups, as presented in Figures 8H, I. The genetic alteration rate in the high-risk group was higher than that in the low-risk subtype (96.35% vs. 92.01%), and we also found that TP53 had a higher mutation frequency in the high-risk group (71% vs. 61%). TP53 and TTN remained the top two genetic alterations in each group. Missense mutations were the most common form of mutation in the two groups.

### Drug sensitivity with prognostic signature

**FIGURE 9:**
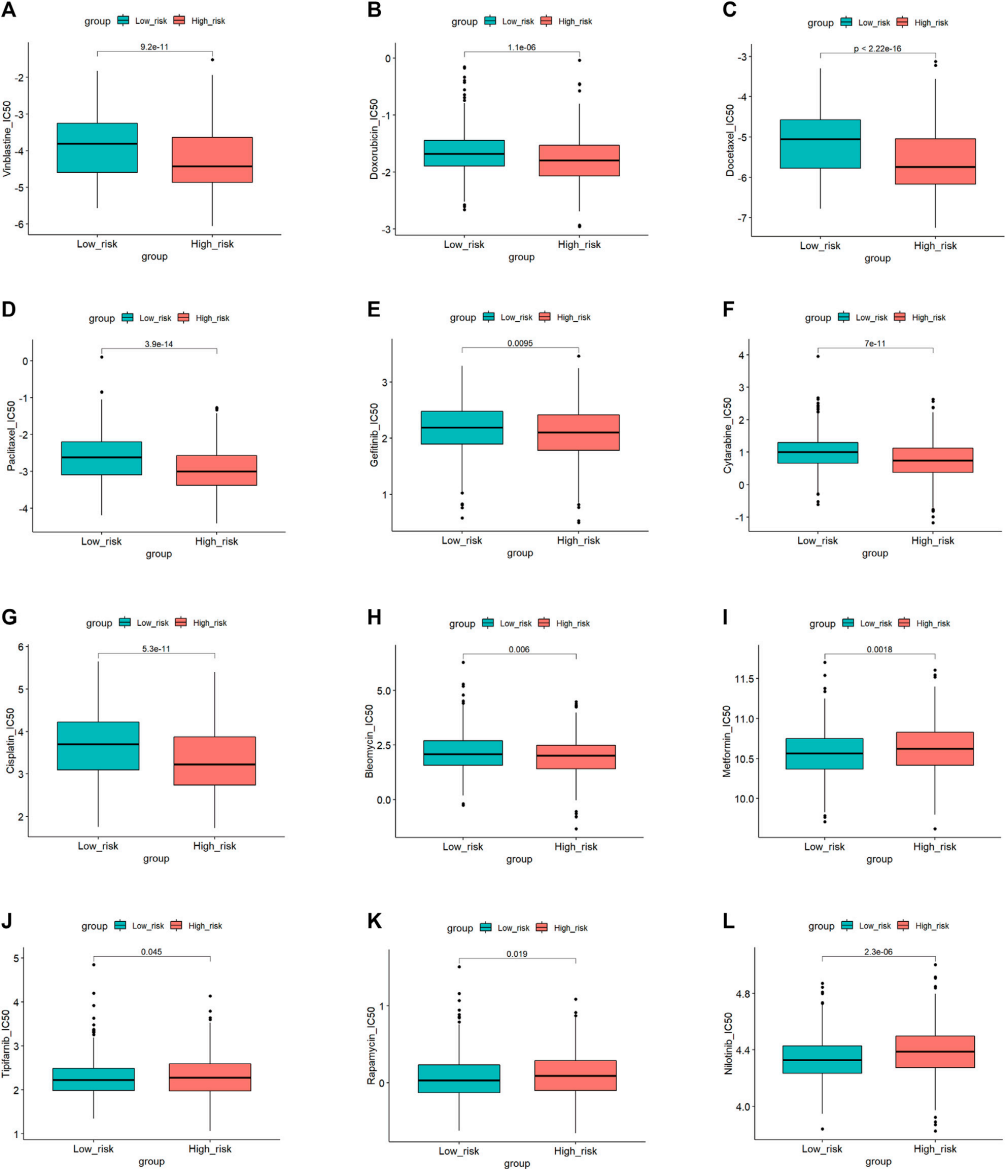
Drugs sensitivity in the high and low-risk group.

To explore the potential application of personalized drug treatment based on our prognostic model, we investigated the IC50 values of different drugs between the two sets. Drug sensitivity tests showed lower IC50 values of cisplatin, doxorubicin, docetaxel, paclitaxel, vinblastine and so on in the high-risk group, which indicated that the medication described above may be effective in high-risk patients. The IC50 values of nilotinib, tipifarnib, rapamycin and metformin were lower in the low-risk group, implying that patients in the low-risk group may benefit from these therapies. Details are shown in Figure 9.

### Experimental and clinical validation

**FIGURE 10:**
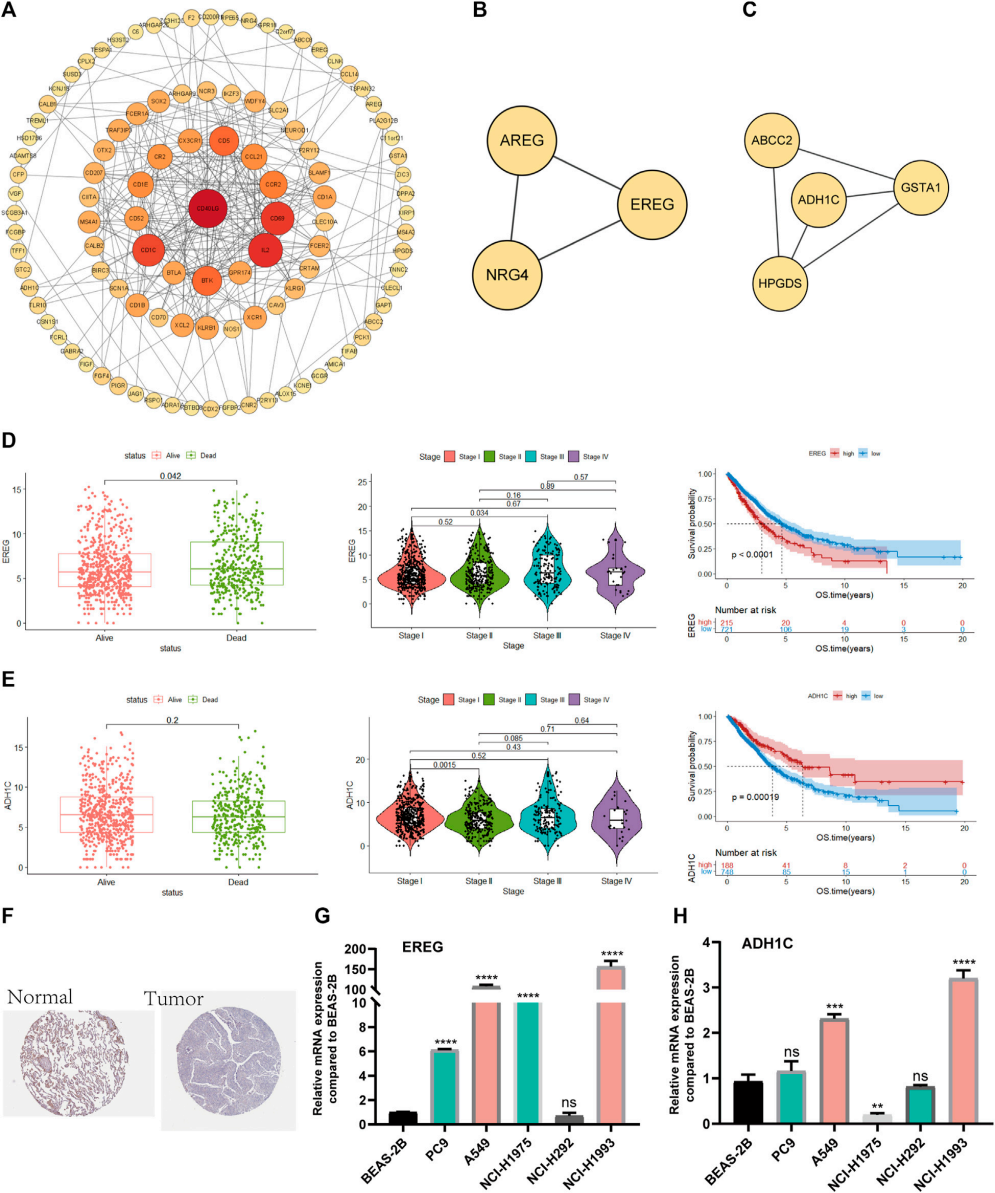
Screening and validation of hub genes **(A)**. PPI network among prognostic genes. **(B, C)** Two modules contained many genes in the PPI network **(D)**. The relationship between survival state, stage and mRNA expression levels of EREG, Kaplan–Meier curves of EREG in OS **(E)**. The relationship between survival state, stage and mRNA expression levels of ADH1C, Kaplan–Meier curves of ADH1C in OS **(F)**. ADH1C protein levels in normal lung and NSCLC were visualized by IHC in HPA. **(G, H)** Quantitative real-time PCR analysis of the mRNA expression levels of EREG and ADH1C in NSCLC cell lines and normal lung epithelial cells.

First, we used the PPI network of the prognostic DEGs from univariate Cox analysis and further visualized it by Cytoscape software (Figure 10A). A total of 7 modules were obtained using MCODE in the PPI network, and two hub genes (EREG and ADH1C) were recognized from two overlapping results between genes of modules and 15 DEGs (Figures 10B, C). Additionally, the variability in survival status and stage was evaluated with differences in hub gene expression. There was a statistically significant higher rate of EREG expression in dead NSCLC patients (*p-value* = 0.042), while a trend toward more ADH1C expression was discovered in surviving patients (Figures 10D1, E1). We also found that EREG was directly proportional to the stage of NSCLC, while the mRNA expression of ADH1C seemed negatively related to the staging system (Figures 10D2, E2). Subsequently, the results of survival analysis showed that EREG was associated with shorter OS and that higher expression of ADHIC was beneficial to survival (*p-value* < 0.05) (Figures 10D3, E3). Similarly, in other datasets ADHIC was related to better survival (*p-value* < 0.05) and the high expression of EREG tends to be worse OS (Supplementary Figure S2).

Moreover, we demonstrated that there was less protein expression of ADH1C in lung tumors than in normal lung tissue based on the HPA database (Figure 10F).

Eventually, PCR experiments were utilized to directly compare the difference in mRNA expression in lung epithelial cells and NSCLC cell lines. EREG demonstrated higher RNA expression in NSCLC than in normal lung epithelial cells, while there was little difference in ADH1C between normal and tumor lung cells, which needs additional experiments for validation (Figures 10G, H).

## Discussion

NSCLC diagnosis and treatment have made breakthroughs in the past few decades due to the ongoing discovery of genomic and TME changes in the pathogenesis of lung cancer. The approach has gradually shifted from chemotherapy drugs that broadly attack tumors toward targeted immunotherapy for precision therapy. Further exploration of the tumor immune microenvironment and identification of meaningful biomarkers are expected to provide a new direction for better patient diagnosis and treatment. In our study, through the ESTIMATE algorithm, we first found that the immune score was connected with the survival of NSCLC patients. Then, we divided the patients into two sets based on the optimum critical point of the immune score and mined the differential genes by the “DESeq2” R package. The prognostic gene signature was established by a series of statistical analyses. The external validation of GSE37745 and GSE31210 illustrated the accuracy of our prognostic model. In addition, relative GSEA, tumor immune, mutation and drug sensitivity analyses were performed between the two risk groups. The experimental validation of the hub gene strengthened our results. In general, the results of our research had a certain constructive impact on the predicted prognosis of NSCLC from the perspective of the tumor immune microenvironment (TIME).

The TIME, containing immune-promoting and immunosuppressive cells and molecules, plays a crucial role in the progression and prognosis of tumors. The immune system undergoes three major changes during the development of tumors: immune surveillance, immune balance and immune destruction (Dunn et al., 2004). It presents the characteristic of the two sides; the immune cells originally presented natural antitumor properties in tumor invasion while uncharacteristically changed into a promoting tumor phenotype during tumor progression, which contributes to immune escape and distant metastasis of malignancy. Currently, the characteristics of the TIME, listed as one of ten tumor characteristics (Hanahan and Weinberg, 2011), play a certain role in predicting both clinical prognosis and the efficacy of chemoradiotherapy (Josefowicz et al., 2012). Therefore, it makes significant sense to analyze the types and distribution of immune cells in the TME and generate an efficient immune evaluation system. Previous studies on the TME of pancarcinoma (mainly gastric cancer) have discovered that patients with high TME scores showed a stronger antitumor immune response, were more likely to benefit from immunotherapy and had better survival outcomes (Fatima et al., 2013). In lung cancer, tumor-infiltrating CD4^+^ T cells play a crucial role in the immune response by allowing CD8^+^ T cells to enter tumor sites and infect mucous membranes to kill tumors; moreover, they are necessary to inhibit tumor angiogenesis (Borst et al., 2018). Other studies have shown that tumor-associated macrophages (TAMs) in the TME are involved in angiogenesis, tumor migration and metastasis and the antitumor immune response, which is related to tumor progression (Qian and Pollard, 2010). Our study applied the ESTIMATE algorithm to NSCLC and found that a high immune score was associated with a better prognosis, which was consistent with previous research.

The gene expression profiles of the two groups with high and low NSCLC immune scores were analyzed and compared, and 1806 DEGs were screened. Subsequently, a 15-gene signature was established through LASSO and Cox analyses, and the risk score of every patient was calculated. To date, a number of gene signatures have been established to predict the survival of NSCLC patients. The research in 2017 identified a tumor immune-associated prognostic gene signature with a precise prediction (AUC = 0.7) in early-stage NSCLC (Li et al., 2017). Liu et al. established a prognostic model that combined molecular biomarkers (TPX2 and MMP12) and several meaningful clinical features and exhibited a higher survival prediction performance (AUC = 0.771) than TNM staging systems in postoperative NSCLC patients (Liu et al., 2018).

However, they rarely translated into medical practice, which may be due to the following three reasons: 1) the gene signature was trained in a cohort with high variance, 2) mRNA microarray data may be measured using diverse experimental methods, and 3) most gene signatures consist of few typical genes and may neglect other potential reasons that seriously reduce their prediction stability and may result in overfitting. Our study found that a high immune score was associated with a better prognosis in NSCLC, and the subsequently constructed immune-related gene signature showed superior prognostic classification ability than other clinical parameters and was further verified in two other GEO datasets. Our study provides an alternative idea for the development of new targeted drugs. The nomogram, a practical tool for assessing the prognosis of malignant patients, has been recognized to be more effective than traditional TNM staging (Diao et al., 2019). Therefore, we combined the risk score and several clinical parameters to draw a nomogram that predicted 1-, 3-, and 5-year survival and exhibited relatively reliable prediction efficiency. It is worth considering that there were more stage I-III NSCLC patients than advanced stage NSCLC patients in the TCGA cohort. Early-stage NSCLC patients tended to have a good prognosis after surgical treatment, and there were more factors related to prognosis. To a certain extent, the lack of specific treatment, clinical characteristics, laboratory indices, and imaging materials in the databases could influence the model accuracy.

The prognostic model established in our research consisted of 15 prognostic DEGs. Furthermore, we selected key prognostic genes from two overlapping results from PPI and 15 DEGs. Two hub genes (EREG and ADH1C) were recognized. Patients with high mRNA expression of EREG tended to have a poor prognosis, while ADH1C was associated with better survival in TCGA. EREG and ADH1C were further verified through RT‒PCR experiments of normal lung epithelium and NSCLC cell lines. Altogether, we determined that EREG and ADH1C likely play significant roles in the process of NSCLC through validation of the TCGA database and RT‒PCR assay.

Epiregulin (EREG), a member of the epidermal growth factor family, combines with ErbB receptors and further contributes to proliferation, inflammation and anti-apoptosis in tumor cells (Riese and Cullum, 2014). Additionally, it promotes the progression of various cancers. Research has revealed that patients with positive EREG are associated with a worse prognosis than those with negative EREG in NSCLC (Zhang et al., 2008). Moreover, Chen et al. (Chen et al., 2019)discovered that acquired resistance to 5-FU in colon cancer can be reversed by inhibiting the miR-215-5p-EREG/TYMS axis. A study (Zhang et al., 2022) in 2022 found that EREG enhanced resistance to chemotherapy in NSCLC by increasing the expression of stemness-associated genes. Notably, the expression of EREG in stromal cells is upregulated and activates several downstream signaling pathways, including the MAPK AKT/mTOR and JAK/STAT pathways, in cancer cells by paracrine signaling, promoting their malignant phenotype and accelerating the progression of cancer (Wang et al., 2022). This discovery reinforces our conclusion that EREG plays a crucial role in NSCLC progression by influencing the TIME.

ADH1C is a member of the ADH family that catalyzes the oxidation of ethyl alcohol to acetaldehyde (a carcinogenic metabolite) and plays a crucial role in the etiology of various cancers. The polymorphism of ADH1C may be a crucial factor in the etiology of oral cancer and genetically determine an individual’s susceptibility (Brocic et al., 2011). A previous study indicated that patients with positive ADH1C have an increased risk of head and neck cancer (Visapaa et al., 2004). In addition, further studies suggested that ADH1C was not linked to HNC (Peters and McClean, 2005). Similar differences were found for colorectal and breast cancer. Bongaerts et al. (2011) considered that the ADH1C genotype and excessive alcohol intake were associated with an increased risk of CRC, while some researchers have suggested that ADH1C expression is reduced during the progression of CRC from early to advanced stages. ADH1C allele mutations were related to an increased breast cancer risk due to alcohol consumption by comparing postmenopausal breast cancer samples with controls (Benzon Larsen et al., 2010). However, one study suggests that ADH1C polymorphisms may not be connected with breast cancer in Caucasians (Wang et al., 2012). The abovementioned inconsistent conclusions regarding the effect of ADH1C on cancer suggested that the mechanisms related to ADH1C may be complex and remain unclear. A recent study showed that the prognosis of NSCLC patients with high ADH1C expression was associated with longer OS. Our research found that ADH1C may be an antitumor factor whose higher expression is associated with a better prognosis. However, reverse protein and mRNA expression requires further experimental verification.

We further explored the potential pathway mechanism between the two subtypes. It is worth noting that the proliferation, differentiation, metastasis of tumor cells and pathways in cytokines and inflammation were enriched in the high-risk group. For example, epithelial-mesenchymal transition (EMT) indicates the malignant process of tumors, allows tumor cells to invade and metastasize, and promotes chemotherapy resistance (Jolly et al., 2019). E2F is essential to cellular homeostasis and plays a role beyond cell cycle regulation, and its dysregulation may lead to cancer progression, including processes such as apoptosis, metabolism and angiogenesis (Kent and Leone, 2019). Tumor necrosis factor-α (TNF-α) is a proinflammatory cytokine involved in normal inflammatory and immune responses. TNF-α receptors exist on various cell surfaces and are divided into two types (TNFR I and TNFR II). The combination of TNF-α and TNFR usually causes inflammation and the occurrence of tumors (Frances, 2009). TNF-α, as a key regulator of the TME, is well-recognized (Wu and Zhou, 2010). Cytokine interactions accounted for a high proportion in GSEA. Cytokines were the key signaling proteins in the TME. TNF and IL-6 can cause disordered cytokine regulation and promote tumor inflammation. IL-10, IL-4, and TGFβ lead to immunosuppression (Propper and Balkwill, 2022). Cytokines and their receptors have been widely studied as tumor targets or therapeutic strategies. These tumor-related pathways were enriched in the set of high-risk patients, and these pathways were closely related to tumor progression and further verified the accuracy of our study.

The establishment of an immune-related prognostic model in our study was validated from multiangle and multiple databases, and the prognostic gene signature demonstrated good prospects in predicting the prognosis of NSCLC patients. Our study further analyzed mutations and drug sensitivity differences between the two risk groups. The results of differentially mutated genes were convenient for subsequent basic experimental research, and the finding of drug sensitivity had potential guiding meaning for the choice of clinical drug. However, there are still some limitations in our research. First, the incomplete and lack of specific treatment, clinical characteristics, laboratory indices, and imaging materials in the TCGA databases could influence the model accuracy, and we could not explore the influence of these factors on NSCLC patients. In addition, we believe that functional experiments should be conducted to confirm the molecular mechanism of the hub genes. Finally, it would be best if the prognostic model was verified in prospective or retrospective clinical trials.

## Conclusion

In conclusion, we have identified an immune-related 15-gene-based prognostic model that presented an accurate prognostic predictive ability in NSCLC patients. The potential mechanisms and chemotherapy-sensitive analyses were also evaluated between the two risk groups, which could provide the basis for subsequent basic and clinical trials.

## Data Availability

The datasets presented in this study can be found in online databases. This data can be found here: The Cancer Genome Atlas (TCGA) (https://portal.gdc.cancer.gov/) and Gene Expression Omnibus (GEO) (https://www.ncbi.nlm.nih.gov/geo/) databases. The accession numbers can be found in the article.
