# Supplementary figures and images for: Integrative bioinformatics approaches to establish potential prognostic immune-related genes signature and drugs in the non-small cell lung cancer microenvironment
Source: Front Pharmacol. 2023 Apr 3;14:1153565. doi: 10.3389/fphar.2023.1153565 (PMC10106634; doi:10.3389/fphar.2023.1153565)

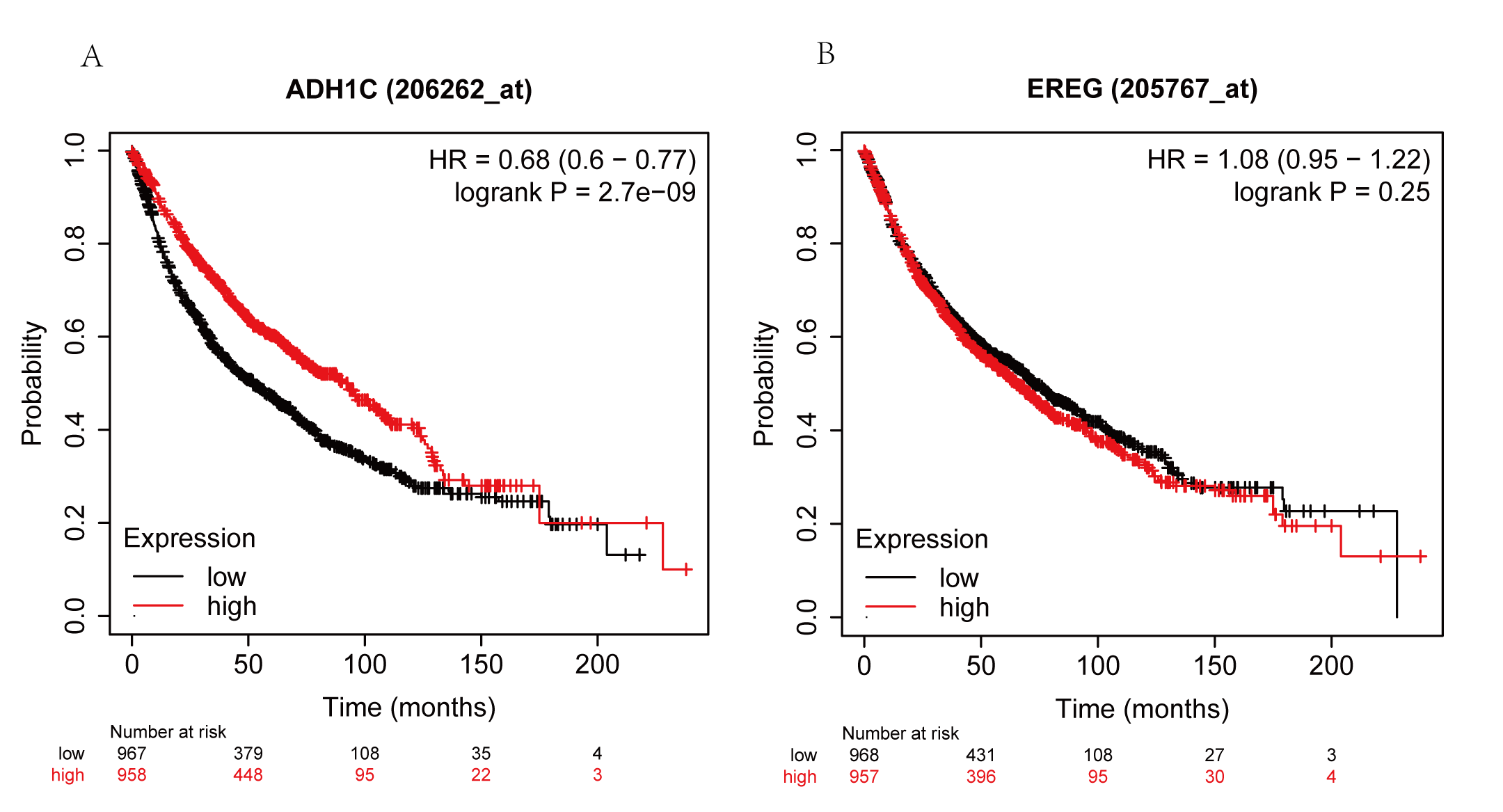

Supplement: Supplementary file 1 [file Image2.TIF]

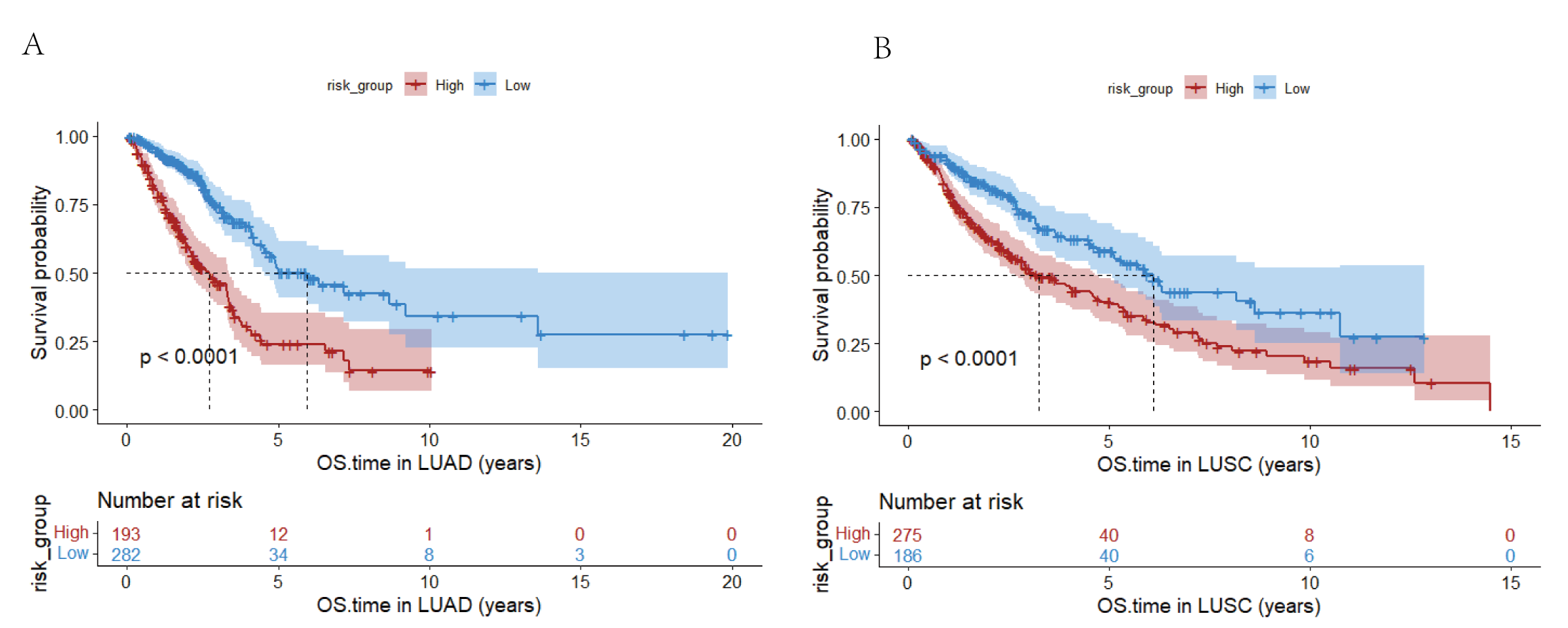

Supplement: Supplementary file 2 [file Image1.TIF]
