# Supplementary material for: Integrative bioinformatics approaches to establish potential prognostic immune-related genes signature and drugs in the non-small cell lung cancer microenvironment
Source: Front Pharmacol. 2023 Apr 3;14:1153565. doi: 10.3389/fphar.2023.1153565 (PMC10106634; doi:10.3389/fphar.2023.1153565)
Supplement: Supplementary file 3 [file DataSheet1.docx]

**Supplementary Materials TABLE 1 ǀ** Results of multivariate cox regression analysis of the OS in TCGA cohort

| **Gene_names** | **Coef** | **HR** | ***P.* value** | **Lower** | **Upper** |
| --- | --- | --- | --- | --- | --- |
| ADH1C | -0.08672 | 0.916929 | 0.001622 | 0.868792 | 0.967734 |
| CPLX2 | 0.149126 | 1.16082 | 1.13E-04 | 1.076186 | 1.252109 |
| CSN1S1 | 0.177855 | 1.194652 | 0.007242 | 1.049224 | 1.360237 |
| NTSR1 | 0.176275 | 1.192766 | 0.012915 | 1.038009 | 1.370595 |
| CDX2 | 0.210644 | 1.234473 | 0.018645 | 1.035777 | 1.471286 |
| ABCC8 | -0.9561 | 0.384387 | 0.001108 | 0.216398 | 0.682788 |
| PCARE | -0.83455 | 0.434068 | 0.03274 | 0.201776 | 0.933781 |
| TNNC2 | -0.10013 | 0.904721 | 0.016508 | 0.833614 | 0.981893 |
| EREG | 0.089497 | 1.093624 | 0.015617 | 1.01709 | 1.175918 |
| MCOLN2 | -0.24566 | 0.782185 | 0.01577 | 0.640756 | 0.95483 |
| CD70 | 0.114536 | 1.121353 | 0.034097 | 1.00863 | 1.246674 |
| GLB1L3 | -0.10665 | 0.898837 | 0.085536 | 0.795944 | 1.015032 |
| CD200R1 | -0.27673 | 0.758256 | 0.087736 | 0.551899 | 1.041772 |
| DEFA3 | 0.634538 | 1.88615 | 0.002867 | 1.24287 | 2.862375 |
| ARL14EPL | 0.359125 | 1.432076 | 0.098948 | 0.934755 | 2.193989 |

Abbreviations: HR, hazard ratio

**Supplementary Materials TABLE 2 ǀ** Relationship between risk score and clinical characteristics of 226 patients in GSE31210 cohort.

| **Characteristic** | **Total** | **High-risk group** | **Low-risk group** | ***χ^2^*** | ***P value*** |
| --- | --- | --- | --- | --- | --- |
|  | **n=226(%)** | **n=113(%)** | **n=113(%)** |  |  |
| **Gender** |  |  |  | 2.152 | 0.182 |
| Male | 121(53.5%) | 55(48.7%) | 66(58.4%) |  |  |
| Female | 105(46.5%) | 58(51.3%) | 47(41.6%) |  |  |
| **Age（years）** |  |  |  | 0.000 | 1.000 |
| <= 65 | 108(47.8%) | 54(47.8%) | 54(47.8%) |  |  |
| >65 | 118(52.2%) | 59(52.2%) | 59(52.2%) |  |  |
| **Stage** |  |  |  | 0.371 | 0.648 |
| Stage I | 168(74.3%) | 82(72.6%) | 86(76.1%) |  |  |
| Stage II | 58(25.7%) | 31(27.4%) | 27(23.9%) |  |  |
| **Smoke** |  |  |  | 0.868 | 0.425 |
| Yes | 111(49.1%) | 59(52.2%) | 52(46.0%) |  |  |
| No | 115(50.9%) | 54(47.8%) | 61(54.0%) |  |  |
| **EGFR mutation** |  |  |  | 6.489 | 0.016 |
| Yes | 127(56.2%) | 54(47.8%) | 73(64.6%) |  |  |
| No | 99(43.8%) | 59(52.2%) | 40(35.4%) |  |  |
| **KRAS mutation** |  |  |  | 1.975 | 0.241 |
| Yes | 20(8.8%) | 13(11.5%) | 7(6.2%) |  |  |
| No | 206(91.2%) | 100(88.5%) | 106(93.8%) |  |  |
| **ALK fusion** |  |  |  | 0.096 | 1.000 |
| Yes | 11(4.9%) | 6(5.3%) | 5(4.4%) |  |  |
| No | 215(95.1%) | 107(94.7%) | 108(95.6%) |  |  |
| **MYC** |  |  |  | 0.573 | 0.615 |
| High | 17(7.5%) | 10(8.8%) | 7(6.2%) |  |  |
| Low | 207(91.6%) | 102(90.3%) | 105(92.9%) |  |  |
| unknown | 2(0.9%) | 1(0.9%) | 1(0.9%) |  |  |

Abbreviations: n, number

**Supplementary Materials TABLE 3 ǀ** Relationship between risk score and clinical characteristics of 196 patients in GSE37745 cohort.

| **Characteristic** | **Total** | **High-risk group** | **Low-risk group** | ***χ^2^*** | ***P value*** |
| --- | --- | --- | --- | --- | --- |
|  | **n=196(%)** | **n=98(%)** | **n=98(%)** |  |  |
| **Gender** |  |  |  | 1.653 | 0.199 |
| Male | 107(54.6%) | 54(55.1%) | 53(54.1%) |  |  |
| Female | 89(45.4%) | 44(44.9%) | 45(45.9%) |  |  |
| **Age（years）** |  |  |  | 0.082 | 0.775 |
| <65 | 94(48.0%) | 48(49.0%) | 46(46.9%) |  |  |
| >=65 | 102(52.0%) | 50(51.0%) | 52(53.1%) |  |  |
| **Stage** |  |  |  | 0.598 | 0.440 |
| Stage I/II | 164(83.7%) | 80(86.1%) | 84(85.7%) |  |  |
| Stage III/IV | 32(16.3%) | 18(18.4%) | 14(14.3%) |  |  |
| **Performance status** |  |  |  | 0.513 | 0.474 |
| 0 | 105(53.6%) | 50(51.0%) | 55(56.1%) |  |  |
| >0 | 91(46.4%) | 48(49.0%) | 43(43.9%) |  |  |
| **Tumor type** |  |  |  | 7.063 | 0.029 |
| LUSC | 66(33.7%) | 39(39.8%) | 27(27.6%) |  |  |
| LUAD | 106(54.1%) | 45(45.9%) | 61(62.2%) |  |  |
| LCLC | 24(12.2%) | 14(14.3%) | 10(10.2%) |  |  |

Abbreviations: n, number
